# Surgery for Esophageal Cancer: Apples and Oranges

**DOI:** 10.4103/1319-3767.77254

**Published:** 2011

**Authors:** Chintamani

**Affiliations:** VMMC, Safdarjang Hospital, New Delhi – 110 023, India. E-mail: chintamani7@rediffmail.com

Sir,

We read with great interest the article by Khan *et al*, regarding predictors of outcome in esophageal cancers. The conclusions of the article, however, only reinforce the significance of some well-known variables. As accepted by the authors themselves, this retrospective study has many limitations. Performance status (Karnowsky or European Co-operative Oncology Group (ECOG) scales) is a very reliable and objective predictor of outcome and one would be keen to know the performance status of patients in both the groups and whether this influenced the type of surgery and preoperative preparation. Although the authors do mention that most patients underwent transhiatal esophagectomy, it would be important to know the type of surgery performed in the “minority.” It is both expected and known that mortality is higher in patients with postoperative morbidity, such as pleural effusion and chylothorax. The authors have attributed the occurrence of pleural effusion to the technique of the surgeon, although this and other complications following esophagectomy are also related to the disease burden.[1–3] It would be of interest to know whether any lymph node dissection was performed and what was the yield in both groups (two-field or three-field surgery)? A major and very important risk factor affecting the outcome of esophageal cancers is the “surgeon” himself.[4] Comparing outcomes in two distinct groups without ensuring that the surgery is performed by the same operating team would be like comparing “apples” to “oranges.”
